# AS-IV Attenuates Oxidative Stress-Induced Apoptosis in Zebrafish via Modulation of the AKT/NRF2/HO-1/Caspase-3 Signaling Axis

**DOI:** 10.3390/molecules30112355

**Published:** 2025-05-28

**Authors:** Jili Dai, Zhizhou E, Yannan Bi, Zetao Yin, Yanfang Wang, Xingyu Wang, Xiaoe Jia, Bo Zou

**Affiliations:** 1 Department of Basic Medicine and Forensic Medicine, Baotou Medical College, Baotou 014040, China; 2 Inner Mongolia Key Laboratory of Hypoxic Translational Medicine, Baotou Medical College, Baotou 014040, China

**Keywords:** AS-IV, NRF2, oxidative stress, network pharmacology, zebrafish

## Abstract

As the primary active component of *Astragalus membranaceus*, Astragaloside IV (AS-IV) is widely recognized in pharmacological research for its multifaceted therapeutic potential, particularly its antioxidative, immunostimulatory, and cardioprotective properties. Oxidative stress is an important mechanism in the induction of many diseases. The present study investigates the antioxidative mechanism of Astragaloside IV in zebrafish, using menaquinone exposure to induce oxidative stress conditions. The findings revealed that AS-IV effectively attenuated oxidative stress-induced mortality and morphological abnormalities in zebrafish. AS-IV exhibited a concentration-dependent protective effect against developmental abnormalities, with progressive reduction in pericardial effusion, body curvature, and growth retardation observed at higher doses. Moreover, AS-IV treatment not only effectively reduced reactive oxygen species (ROS) accumulation and attenuated oxidative DNA damage but also significantly decreased apoptosis in the cardiac region of zebrafish embryos under oxidative stress conditions. Western blot analysis revealed that AS-IV treatment significantly reduced the protein levels of both Cleaved Caspase-3 and γ-H2AX, indicating its ability to inhibit DNA damage-induced apoptosis. AS-IV mediates its antioxidant defense mechanisms through the activation of the nuclear factor erythroid 2-related factor 2 (NRF2) signaling pathway, inducing the significant upregulation of cytoprotective enzymes. This molecular mechanism underlies the observed phenotypic improvements in oxidative stress-related damage. Upstream analysis demonstrated that AS-IV activates NRF2 primarily through protein kinase B (AKT/PKB) pathway modulation, independent of KEAP1 regulation. Comprehensive mechanistic analysis reveals that Astragaloside IV mitigates oxidative stress-induced apoptosis in zebrafish through coordinated regulation of the AKT/NRF2/HO-1/Caspase-3 signaling axis.

## 1. Introduction

Astragali Radix (*Astragalus membranaceus* (Fisch.) Bge mongholicus (Bge.) Hsiao) has a long history clinically used as a traditional Chinese medicine [1]. The medicinal component of Astragali Radix is derived from its dried root, which is utilized for the improvement and treatment of various illnesses, serving both as a medicinal remedy and a food ingredient [2]. Astragali Radix mainly contains polysaccharides, a variety of flavonoids, saponins, alkaloids, and other active ingredients. The pharmacological effects of Astragali Radix include enhancement of immune function, antioxidant activity, radioprotection, and anticancer properties [3]. AS-IV is the core active ingredient of Astragali Radix membranaceus. Pharmacological investigations have demonstrated that AS-IV exhibits multifaceted therapeutic properties, including significant antioxidant, anti-inflammatory, and immunomodulatory activities [4]. Currently, positive therapeutic effect of AS-IV is reported in cardiovascular diseases, diabetes, cancer, and other diseases [5,6,7,8].

Oxidative stress (OS) is an important mechanism to induce many diseases. The essence of this is an imbalance between the production of oxidants and the antioxidant protection mechanisms, and this imbalance has the potential to cause damage to the organism [9]. Oxidative stress causes disease by damaging biomolecules such as DNA, proteins, and membrane lipids, triggering cellular damage. The increase in free radicals, which is due to biological oxidative processes, leads to structural and functional damage to intracellular proteins, membrane damage due to the peroxidation of membrane lipids, the modification of nucleic acid bases, and chromosomal alterations (including DNA single- and double-stranded breaks and cross-linking of DNA to proteins) and ultimately leads to cell death and oxidative damage to cellular structures and components [10]. Increased reactive oxygen species (ROS) due to oxidative stress is known to be strongly associated with pathological processes in a variety of diseases, such as metabolic syndrome, cancer, cardiovascular diseases, rheumatoid arthritis, neurodegenerative diseases, and pre-eclampsia [11,12,13,14,15]. These findings underscore the critical importance of developing effective antioxidant therapies for both prophylaxis and management of oxidative stress-related disorders. The antioxidant activity of AS-IV significantly reduces oxidative damage to cellular biomolecules, including DNA strand breaks, protein carbonylation, and lipid peroxidation, leading to attenuated disease progression [16,17]. Therefore, research into the antioxidant properties of AS-IV is receiving a lot of attention.

The zebrafish (Danio rerio) was employed as the model organism in this study. The zebrafish exhibits remarkable conservation with humans across multiple biological systems, including tissue architecture, developmental processes, and physiological systems (digestive and circulatory). Notably, their molecular components demonstrate high evolutionary conservation, with approximately 70% of human genes having at least one zebrafish ortholog and 80% of disease-associated human proteins having functional zebrafish counterparts [18,19].

This study establishes zebrafish as a novel model system for investigating the antioxidant mechanisms of AS-IV, representing the first application of this vertebrate model in AS-IV research. The effects of AS-IV on menaquinone-induced oxidative stress, such as mortality, malformation rate, and pericardial edema were evaluated. In addition, the mRNA and protein expression levels of genes related to antioxidant capacity, DNA damage and apoptosis were analyzed. Conclusively, our study provides valuable results for the antioxidant properties of AS-IV in zebrafish and set up a theoretical basis for the clinical application of AS-IV.

## 2. Results

### 2.1. Menaquinone Triggered Oxidative Stress and Developmental Abnormalities in Zebrafish Embryos and Larvae

**Figure 1:**
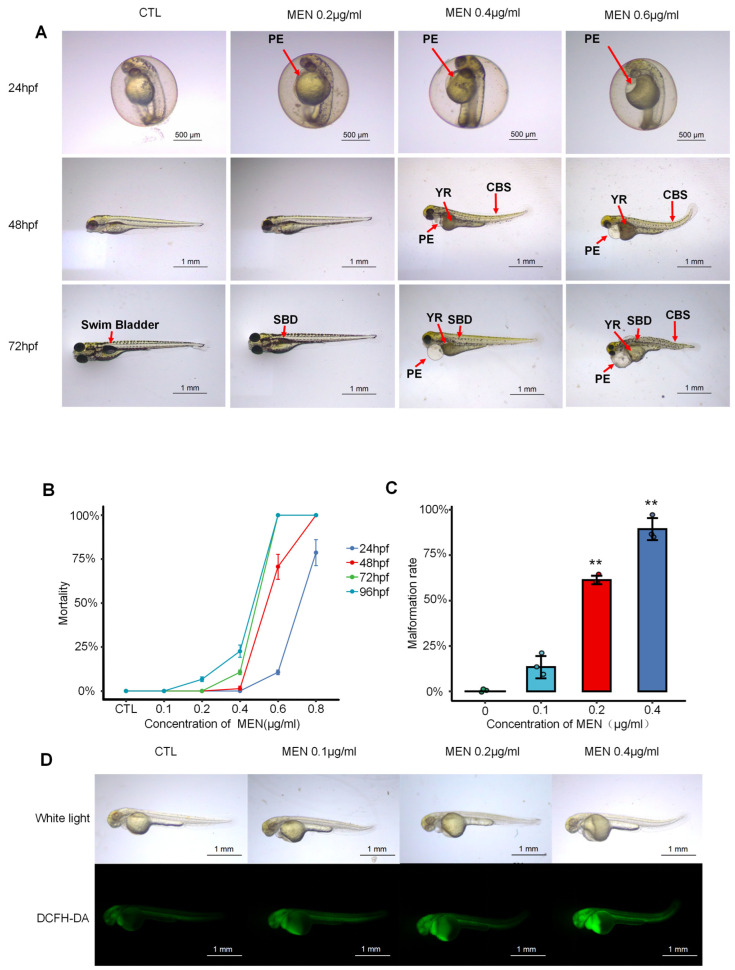
Developmental abnormalities in zebrafish phenotypes due to menaquinone. (**A**) The phenotype of developmental abnormalities in zebrafish caused by menaquinone; (**B**) effects of menaquinone on zebrafish mortality; (**C**) effects of 72 hpf menaquinone on zebrafish deformity rates; (**D**) effects of menaquinone on zebrafish reactive oxygen species accumulation. (PE: pericardium edema; YR: yolk retention; SBD: swim bladder deficiency; CBS: curved body shape). Note: ** *p* < 0.01 versus the CTL group. In this and the subsequent figures, CTL signifies vehicle control (0.1% DMSO).

Developmental toxicity was observed in zebrafish embryos and larvae exposed to different concentrations (0, 0.2, 0.4, 0.6 μg/mL) of menaquinone (MEN) at 24, 48, and 72 h post fertilization (hpf). As shown in Figure 1A, the pericardial edema area of zebrafish embryos gradually increased at 24 hpf. Developmental defects showed temporal progression across MEN concentrations: at 48 hpf, larvae displayed early teratogenic effects including tail curvature, fin growth retardation, and yolk absorption delay, while at 72 hpf, observations revealed advanced pathologies—notably expanded pericardial sacs, absent swim bladders, and significant body shortening.

The mortality rate increased after MEN treatment in a dose-dependent and time-dependent manner with a 100% mortality rate at 72 hpf, 0.6 μg/mL, as shown in Figure 1B. As the concentration of MEN increased, so too did the deformity rate of zebrafish (Figure 1C). Indeed, deformity rates were found to be significant at concentrations above 0.1 μg/mL, with a 90% deformity rate being reached at a concentration of 0.4 μg/mL after 72 h had passed. The most common phenotype in menaquinone-treated embryos is pericardial effusion, suggesting that menaquinone is cardiotoxic. DCFH-DA (2′-7′dichlorofluorescein diacetate) staining showed that MEN dose-dependently elevated ROS levels, with striking cardiac-specific accumulation (Figure 1D). Fluorescence intensity increased linearly from 0.2 to 0.4 μg/mL MEN. Based on preliminary dose–response analyses, we established 0.6 μg/mL MEN as the optimal concentration for mortality studies, while 0.4 μg/mL was selected for other phenotypic assessments. These results demonstrate that MEN induces oxidative stress and developmental abnormalities in zebrafish embryos and larvae.

To assess the potential toxicity of AS-IV, changes in deformity and mortality rates in zebrafish were monitored across a concentration range of 0–500 μg/mL. Based on the results of mortality statistics in Figure S1A and deformity rate experiments in Figure S1B combined with the drug effects, 36, 72, and 108 μg/mL were ultimately selected as the therapeutic concentrations of AS-IV for mitigating menaquinone-induced oxidative damage in zebrafish.

### 2.2. Antioxidant Properties of Astragaloside IV

**Figure 2:**
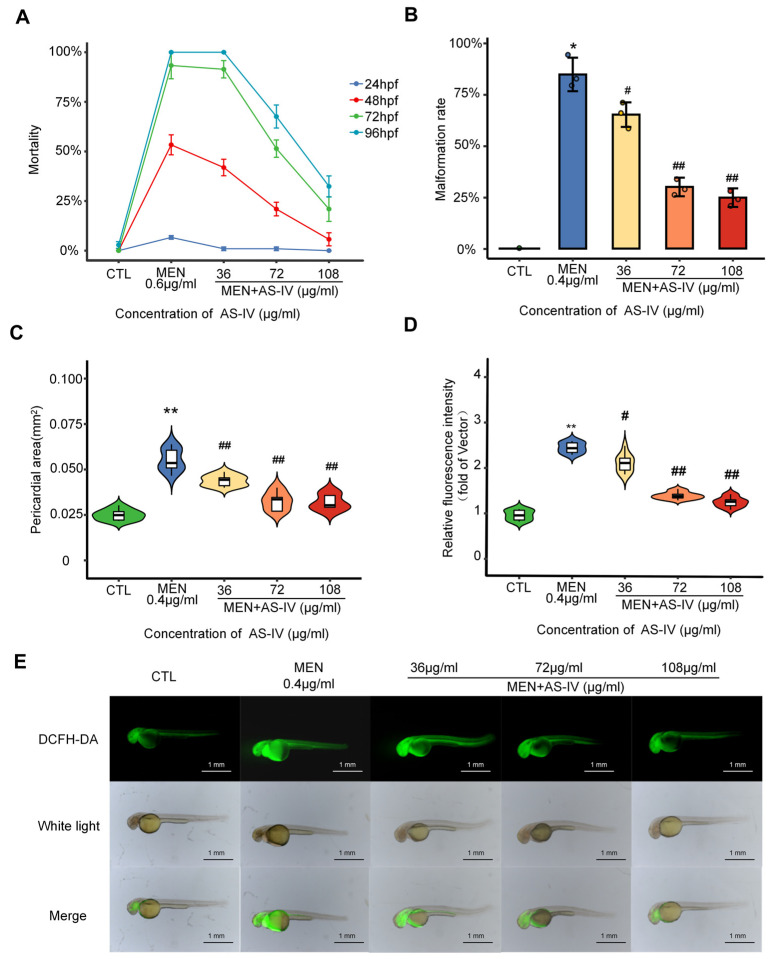
Antioxidant effect of AS-IV. (**A**) AS-IV inhibits increased mortality in an oxidative stress model; (**B**) AS-IV reduces deformity rate in zebrafish; (**C**) AS-IV inhibits the area of pericardial edema; (**D**) fluorescence intensity statistics of DCFH-DA reactive oxygen content assay; (**E**) AS-IV inhibits ROS accumulation in zebrafish. Note: * *p* < 0.05 or ** *p* < 0.01 versus the CTL group. # *p* < 0.05 or ## *p* < 0.01 versus the MEN group.

AS-IV is the main active ingredient of Astragalus and previous studies have shown its antioxidant effects. In the present study, the antioxidant mechanism of AS-IV was investigated using a concentration gradient of (36, 72, and 108 µg/mL), as detailed in Figure S1. With the increase in AS-IV concentration, the mortality rate and malformation rate result from oxidative stress were a significantly decreased in dose-dependent manner (Figure 2A,B). In a variety of dysmorphic developmental phenotypes, cardiac damage was significantly reduced (Figure 2C). ROS production was assayed using DCFH-DA, and it was observed that zebrafish reactive oxygen species accumulation decreased with the elevated AS-IV concentration (Figure 2D,E). In conclusion, AS-IV can alleviate damage caused by a variety of oxidative stresses in zebrafish, but the molecular mechanisms must still be investigated.

### 2.3. Astragaloside IV Suppresses Toxicity in Developing Hearts

**Figure 3:**
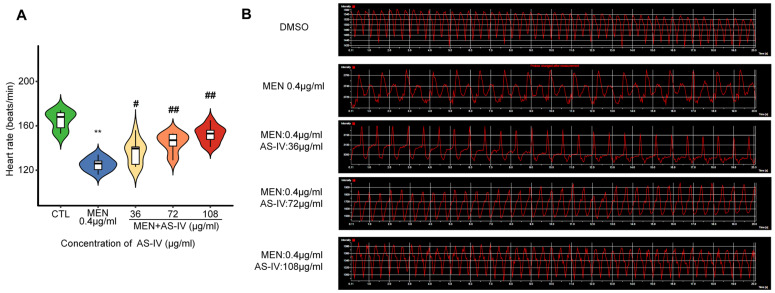
AS-IV treatment of oxidative stress-induced cardiac injury. (**A**) Decrease in heart rate in AS-IV-treated oxidative stress model; (**B**) electrocardiogram of AS-IV intervention oxidative stress model. Note: ** *p* < 0.01 versus the CTL group. # *p* < 0.05 or ## *p* < 0.01 versus the MEN group.

The heart was found to be the organ with the highest accumulation of ROS measured by DCFH-DA (Figure 2D). The heart rate of zebrafish was measured at 72 hpf after AS-IV treatment. Figure 3A shows that the heart rate of juvenile zebrafish in the oxidative stress modeling group decreased significantly compared with that of the control group and then recovered significantly after AS-IV added in a dose-dependent manner. According to Figure 3B, the electrocardiogram conducted by laser confocal reveals the occurrence of heart rate irregularity in the modeling group. under AS-IV treatment, the heart rate gradually recovered to be similar to that of the control group, and the ECG ripples gradually returned to normal. Compared with the oxidative stress modeling group, the heart rate recovered and the pericardial area was significantly reduced in the AS-IV-treated group, which further demonstrated the antioxidant function of AS-IV.

### 2.4. Network Pharmacology Analysis of the Antioxidant Targets of Astragaloside IV

**Figure 4:**
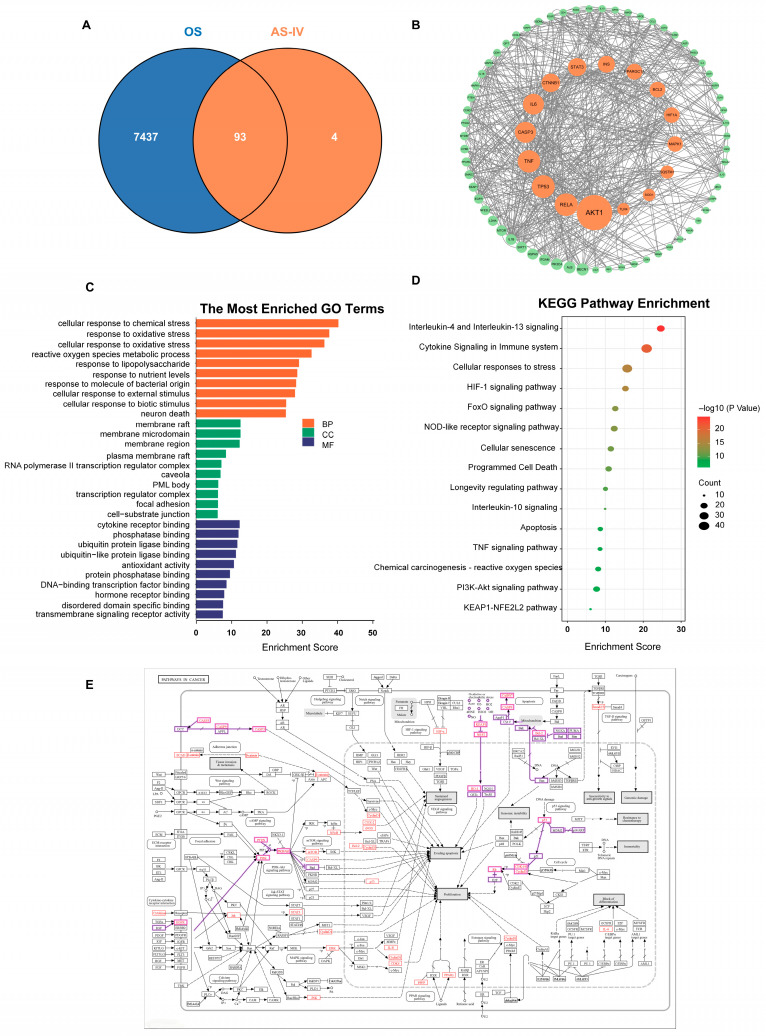
AS-IV antioxidant mechanisms network pharmacology analysis. (**A**) Venn diagram of AS-IV effective targets and oxidative stress targets; (**B**) results of PPI network analysis of AS-IV interfering with oxidative stress targets; (**C**) GO enrichment analysis (the top 20 terms of BP, CC, and MF enrichment analysis were shown in orange, blue, and blue bars, respectively); (**D**) bubble plot of antioxidant pathway enrichment analysis of 93 targets (Top 15 results); (**E**) AS-IV antioxidant major gene KEGG genes mapping.

To further analyze the antioxidant mechanism of AS-IV, network pharmacology techniques were used and 100 major targets of AS-IV were obtained using PubChem and HERB databases. The GeneCards database was used to obtain 10,370 oxidative stress (OS)-related disease targets, and 7530 disease targets were obtained by screening using correlation score ≥ 1 as an indication. VENNY2.1 was used to match 100 AS-IV active ingredient targets with 7530 oxidative stress targets to obtain 93 AS-IV antioxidant targets and Venn diagrams were plotted (Figure 4A). The 93 common targets of Astragalus and oxidative stress were imported into the STRING database, and the species was limited to ‘homo sapiens’, and the highest confidence level (confidence ≥ 0.1080) was used as the index for the screening. The recently formed protein–protein interaction (PPI) network comprised 79 nodes, featuring 1916 edges and an average node connectivity of 48.5%. The network phase connection was stable with high confidence, and the PPI network was beautified using cytoscape software 3.9.1 (Figure 4B). Gene Ontology Enrichment Analysis (GO) and pathway enrichment analyses were performed through the DAVID database for 93 targets related to oxidative stress by active Astragalus ingredients, which were screened with *p* < 0.05 as the index screen. The entries were ranked according to −lgP size, and the top 15 entries were visualized using the Microbiology Letter website (Figure 2C,D). The KEGG database was applied for gene mapping, and genes with changes were marked in red (Figure 4E). Combining the results of multiple analyses, PI3K-AKT signaling pathway, DNA damage leading to apoptosis and the KEAP1-NRF2 signaling pathway will be the focus of future research.

### 2.5. The Impact of AS-IV on Apoptosis and DNA Damage Triggered by Oxidative Stress

**Figure 5:**
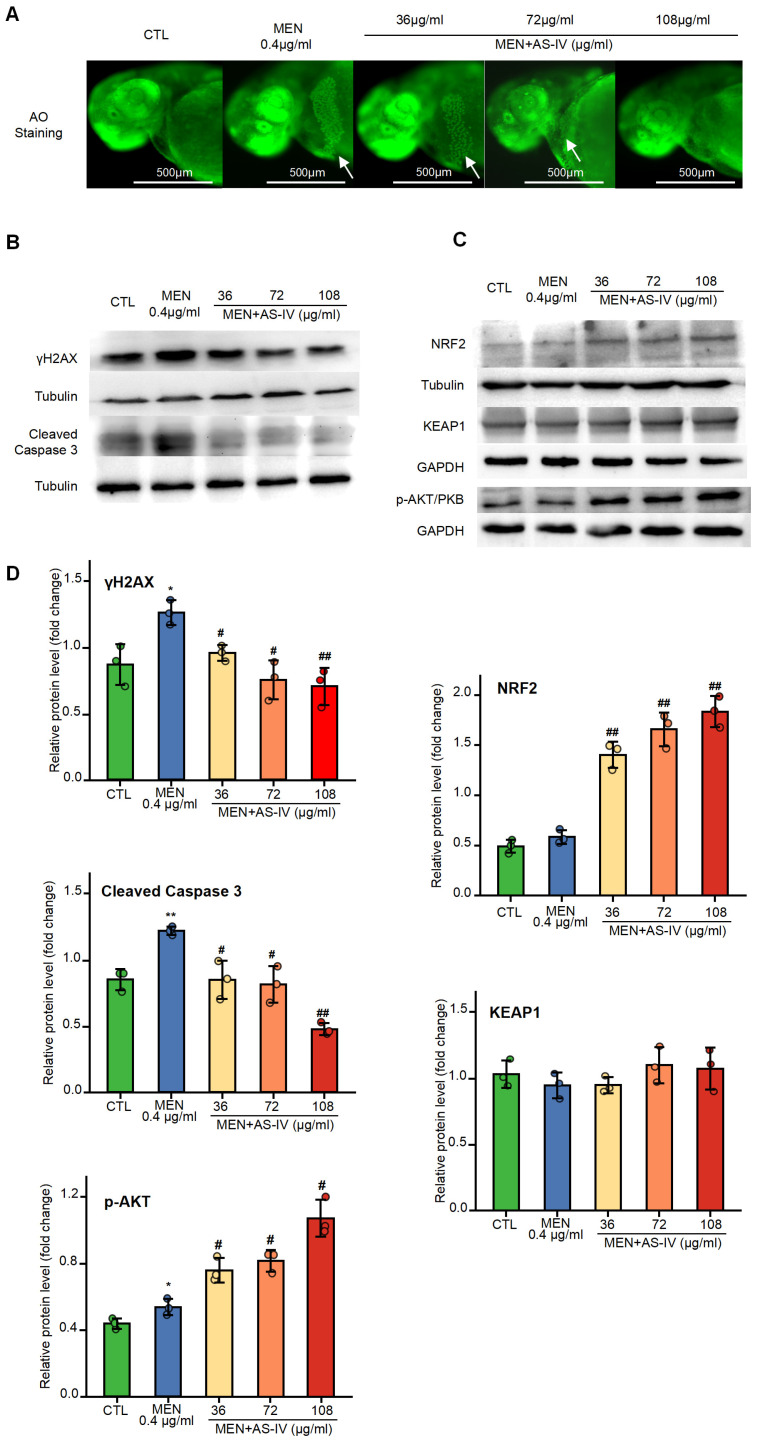
Experimental validation of the antioxidant molecular mechanism of AS-IV. (**A**) Inhibition of zebrafish apoptosis by AS-IV detected by using AO staining (arrowheads point to apoptotic cells); (**B**) assay of γH2AX and cleaved caspase-3 content via Western blot analysis showed that AS-IV inhibited oxidative stress-induced DNA damage and cell apoptosis; (**C**) Western blot analysis results showed that AS-IV increased NRF2 accumulation and AKT/PKB phosphorylation without affecting KEAP1 levels; (**D**) results of the relative protein level. * *p* < 0.05 or ** *p* < 0.01 versus the CTL group. # *p* < 0.05 or ## *p* < 0.01 versus the MEN group.

In order to validate the results of the network pharmacological analysis, we performed an apoptosis assay, in which zebrafish larvae were stained with Acridine Orange 10-nonyl bromide (AO), and only cells that underwent apoptosis were stained. Zebrafish embryos were stained and observed under a fluorescence microscope to identify the apoptosis inhibition role of AS-IV (Figure 5A). Embryos treated with MEN modeling do produce a large number of apoptotic cells in the cardiac region; however, the amount of apoptosis cells was reduced under increasing AS-IV concentration. These results correlate with the development of pericardial edema, suggesting that the pericardial and cardiac regions are the predominant phenotypes in the oxidative stress model and that AS-IV functions as an inhibitor of apoptosis. Cleaved caspase-3 protein expression was quantified by Western blot analysis at 72 hpf (Figure 5B). Result shown that AS-IV could inhibit the accumulation of activated caspase-3, which further demonstrated that AS-IV could inhibit apoptosis. The expression of γH2AX also decreased with the elevation of AS-IV at 72 hpf (Figure 5B), suggesting that AS-IV can inhibit DNA damage by reduced reactive oxygen species. AS-IV reduces DNA damage as the reason for decreased cell apoptosis.

### 2.6. The Effects of AS-IV on Antioxidant Gene Expression

**Figure 6:**
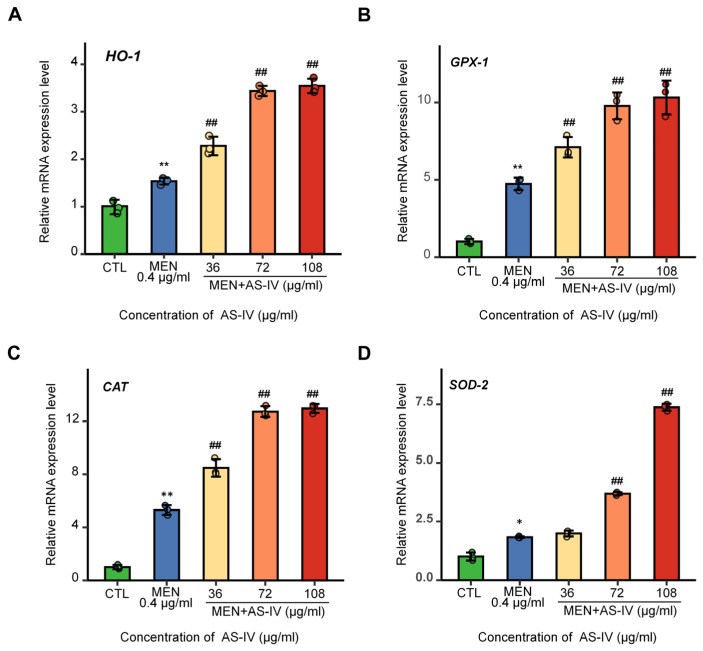
Expression of antioxidant enzymes of AS-IV. (**A**–**D**) The results of qRT-PCR assay showed that AS-IV activated the expression of antioxidant genes HO-1, Gpx-1, CAT, and SOD-2. * *p* < 0.05 or ** *p* < 0.01 versus the CTL group. ## *p* < 0.01 versus the MEN group.

To elucidate the antioxidant mechanism of AS-IV, we systematically evaluated protein expression profiles via Western blot analysis and the transcriptional regulation of antioxidant genes through quantitative real-time PCR (qPCR) at 72 hpf. The protein content of nuclear factor erythroid 2-related factor 2 (NRF2) was detected at 72 hpf by Western blot, and the results showed that AS-IV caused a large accumulation of NRF2 protein. The accumulation of NRF2 increased with increasing AS-IV concentration (Figure 5C). The regulation of the NRF2 pathway was examined and found that there was no significant change in the level of Kelch-like ECH-associated protein 1 (KEAP1), We examined the regulation of the NRF2 pathway and found that the level of Kelch-like ECH-associated protein 1 (KEAP1) showed no significant change, compared to the control group (*p* > 0.05), while the level of phosphorylated protein kinase B (AKT/PKB) was significantly increased, suggesting that AS-IV can activate cellular antioxidant through the activation of AKT/PKB, thus increasing the stability of NRF2 (Figure 5C,D). Subsequently, the expression of downstream antioxidant enzymes was analyzed by qPCR, including Heme oxygenase-1 (HO-1), Superoxide Dismutase-2 (SOD-2), Catalase (CAT), and Glutathione Peroxidase-1 (GPX-1). The results showed that AS-IV induced the expression of these genes in a dose-dependent manner (Figure 6). In conclusion, AS-IV can activate the antioxidant system of zebrafish by activating AKT/PKB to promote the accumulation of NRF2 protein, which in turn enhances its scavenging capacity for ROS. Consistent with network pharmacology predictions, AS-IV exerts its effects by regulating three key pathways: PI3K-AKT signaling, DNA damage-induced apoptosis, and KEAP1-NRF2 signaling.

## 3. Discussion

The zebrafish shares approximately 80% genetic similarity with humans, and its KEAP1-NRF2 system is highly homologous to that of mammals [19,20]. The zebrafish *NRF2* and *KEAP1* genes were first cloned in 2002, with studies demonstrating their structural resemblance to mammalian orthologs [21]. Studies using nrf2 mutant zebrafish models have confirmed the protective role of NRF2 against xenobiotic exposure and oxidative stress [22,23]. Notably, the array of NRF2-regulated target genes in zebrafish exhibits evolutionary conservation. Key components including detoxification pathway proteins, antioxidant proteins, proteasome subunits, and enzymes involved in the pentose phosphate pathway are all regulated by the NRF2 system in zebrafish [23,24]. Therefore, zebrafish serve as a valuable model for studying antioxidant mechanisms, showing significant parallels with those observed in mammals.

The use of menaquinone to create a zebrafish oxidative stress model to simulate radiation injury has been previously reported, but that article did not perform in-depth experiments on the menaquinone zebrafish oxidative stress model [25,26]. In this experiment, a model of oxidative stress in zebrafish induced by menaquinone was established with menaquinone. The oxidative stress injury in the model was mainly manifested as pericardial edema in zebrafish. DCFH-DA assays demonstrated that menaquinone significantly increased ROS accumulation in zebrafish, with the highest fluorescence intensity observed in the cardiac region. This finding correlated with the observed pericardial edema phenotype [27].

Oxidative stress causes DNA damage and can trigger multiple forms of cell death, including apoptosis, necrosis, and ferroptosis [28]. Damage-associated molecular patterns (DAMPs), such as HMGB1 and ATP, are endogenous molecules released from injured or dying cells via passive leakage or active secretion. These molecules bind to pattern recognition receptors (e.g., TLRs, NLRs), initiating a proinflammatory cascade characterized by the production of cytokines like IL-1β and TNF-α. Such cascading reactions ultimately exacerbate tissue and organ damage [29,30]. Clinical trials have identified chronic inflammatory diseases as cardiovascular risk factors [31], while recent studies demonstrate that inflammatory cells promote vascular oxidative stress [32]. AS-IV disrupts the vicious cycle of oxidative stress and inflammation through synergistic antioxidant, anti-inflammatory, and anti-cell death mechanisms, significantly ameliorating cardiovascular pathology in animal models [33,34,35]. Its low toxicity and multi-target efficacy highlight its potential as a therapeutic candidate for cardiovascular diseases, though further clinical studies are required to confirm its safety and efficacy.

The most obvious developmental toxicity phenotype found by the oxidative stress model was pericardial edema, as shown in Figure 1A. A physiological abnormality associated with pericardial edema is a significant decrease in heart rate in zebrafish, characterized by bradycardia (Figure 3A,B). This phenomenon is likely attributed to the onset of apoptosis triggered by the excessive accumulation of ROS in the zebrafish heart (Figure 2E and Figure 5A). It can be argued that apoptosis caused by reactive oxygen species damage to intracellular macromolecular structures is a central factor in the development of pericardial edema. Network pharmacological analysis revealed that AS-IV modulates three key pathways: DNA damage-induced apoptosis; the PI3K-AKT signaling pathway; the KEAP1-NRF2 signaling pathway. As shown in Figure 2E, AS-IV significantly inhibited the accumulation of reactive oxygen species in zebrafish. Figure 5A shows that AS-IV can inhibit the occurrence of apoptosis in zebrafish. AS-IV not only suppressed areas of pericardial edema in zebrafish, but it also treated heart rate slowing and arrhythmias in zebrafish. It suggests that AS-IV can alleviate a series of heart injury-related phenotypes by inhibiting the accumulation of reactive oxygen species.

**Figure 7:**
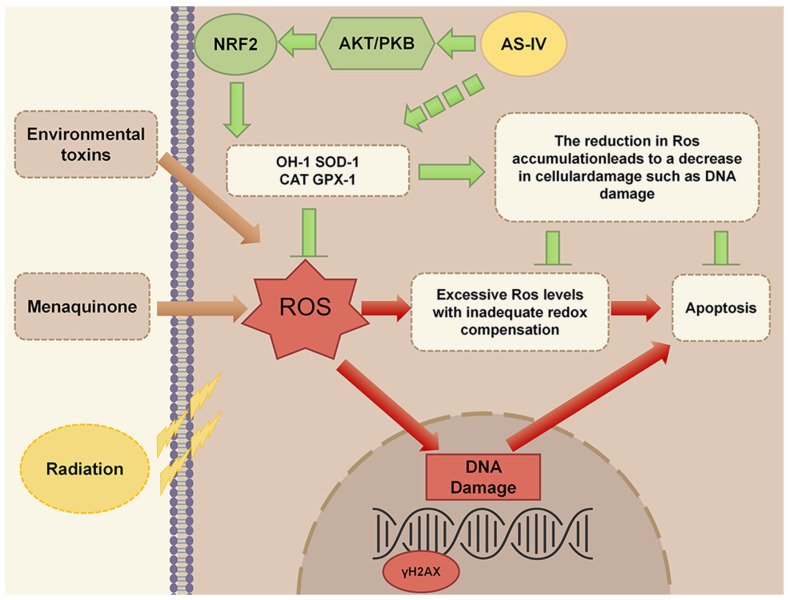
The molecular mechanism through which AS-IV suppresses cell apoptosis triggered by ROS involves the mediation of NRF2 accumulation.

The expression of these related genes was examined at both RNA and protein levels. Firstly, the damage of oxidative stress to DNA, the most important macromolecule substance of the cell, was detected, and the results showed that γH2AX in the oxidative stress model was significantly accumulated, indicating a significant increase in DNA damage relative to the control [36], whereas the accumulation of γH2AX was significantly decreased by treatment with AS-IV, which resulted in a reduction in DNA damage (Figure 5B). Subsequently, for the apoptosis process detection, the activation size subunit of the apoptosis execution protein caspase-3 was detected using WB, and the results showed that AS-IV significantly inhibited the activation of caspase-3, which in turn inhibited cell apoptosis (Figure 5B). Finally, NRF2 accumulation and regulation were examined, and WB results showed that AS-IV significantly increased NRF2 accumulation by a mechanism accomplished by regulating activation AKT/PKB but not by regulating KEAP1 [37,38,39] (Figure 5C). The qRT-PCR results showed the expression of the enzymes gene *HO-1*, *SOD-2*, *CAT*, and *GPX-1* [40,41], which are antioxidants in the zebrafish organism (Figure 6). This revealed that in zebrafish, AS-IV activates antioxidant enzyme content by promoting NRF2, thereby inhibiting reactive oxygen species accumulation to alleviate cellular DNA damage and ultimately inhibit apoptosis, as shown in Figure 7. Reduced apoptosis inhibits a developmental toxicity phenotype represented by pericardial edema.

## 4. Materials and Methods

### 4.1. Chemicals

AS-IV (CAS No.84687-43-4, purity > 98%) was procured from Shanghai Yuanye Biotechnology Co., Ltd. (Shanghai, China). AS-IV was dissolved in dimethyl sulfoxide (DMSO) to prepare a 20 mM storage solution, stored at −20 °C. Menadione (CAS No. 57414-02-5, purity > 98%) was purchased from Shanghai Yuanye Biotechnology Co., Ltd. (Shanghai, China). AS-IV (CAS No.84687-43-4, purity > 98%) was procured from Shanghai Yuanye Biotechnology Co., Ltd. (Shanghai, China). AS-IV was dissolved in dimethyl sulfoxide (DMSO) to prepare a 20 mM storage solution, stored at −20 °C. Menadione (CAS No. 57414-02-5, purity > 98%) was purchased from Shanghai Yuanye Biotechnology Co., Ltd. (Shanghai, China). Before the experiment, the working solution was prepared by diluting the storage solution with egg water. 2′-7′dichlorofluorescein diacetate (DCFH-DA) (CAS No. 4091-99-0, purity > 98%) was procured from Shanghai Yuanye Biotechnology Co., Ltd. (Shanghai, China). A stock solution of 2 mg/mL was prepared using DMSO for solubilization and subsequently diluted with egg water to obtain the working solution. Acridine Orange 10-nonyl bromide (AO) (CAS No.75168-11-5, purity > 98%) was procured from Shanghai Yuanye Biotechnology Co., Ltd. (Shanghai, China). All chemicals and reagents used in this study were of analytically pure grade.Before the experiment, the working solution was prepared by diluting the storage solution with egg water. 2′-7′dichlorofluorescein diacetate (DCFH-DA) (CAS No. 4091-99-0, purity > 98%) was procured from Shanghai Yuanye Biotechnology Co., Ltd. (Shanghai, China). A stock solution of 2 mg/mL was prepared using DMSO for solubilization and subsequently diluted with egg water to obtain the working solution. Acridine Orange 10-nonyl bromide (AO) (CAS No.75168-11-5, purity > 98%) was procured from Shanghai Yuanye Biotechnology Co., Ltd. (Shanghai, China). All chemicals and reagents used in this study were of analytically pure grade.

### 4.2. Rearing Zebrafish and Collecting Samples

The AB strain of zebrafish was acquired from the National Zebrafish Resource Center (NZRC). Zebrafish were maintained in a recirculating aquatic housing system under controlled environmental conditions: pH 7.0 ± 0.1, temperature 28.5 ± 0.5 °C, and a 12 h light/dark photoperiod. For mating protocols, sexually mature fish were transferred to specialized breeding tanks at a female-to-male ratio of 1:2, separated by transparent partitions during nocturnal acclimatization. Synchronized spawning was initiated by partition removal at 08:00 h the following morning, with embryo collection commencing 2–4 h post-spawning. Fertilized embryos were subsequently maintained in a temperature-controlled incubator (28.5 °C) containing standard egg water (0.2% *w/v* instant ocean salt in deionized water). The zebrafish used in this study were maintained in the model animal platform at the Center of Translational Medicine in Baotou Medical College. This study was reviewed and approved by the Experimental Animal Ethics Committee of the Baotou Medical College, Inner Mongolia University of Science and Technology, Inner Mongolia China (approval no.202418).

### 4.3. Modeling Oxidative Stress and Treatment with AS-IV

Embryos were examined under a microscope (Nikon, SMZ18, Tokyo, Japan) at 8 h post fertilization (hpf) to select those with normal development for subsequent experiments. Embryos were distributed into 6-well plates with 25 juveniles per well containing 3 mL of solution. Zebrafish embryos were exposed to different concentrations of Menadione (0.10 µg/mL, 0.20 µg/mL, 0.40 µg/mL, 0.60 µg/mL, 0.80 µg/mL) dilutions at 8 hpf, and zebrafish embryos were treated with 0.1% DMSO as a control (CTL), with three technical parallels and biological parallels for each concentration.

Wild-type zebrafish embryos (AB strain) at 8 h post-fertilization (hpf) were systematically collected and distributed into 6-well culture plates. Experimental groups consisting of 3 embryos per well were maintained in 3 mL of egg water supplemented with 0.4 μg/mL Menadione combined with ascending concentrations of AS-IV (36, 72, and 108 μg/mL). A parallel control group received 0.1% DMSO vehicle solution. Developmental morphology was longitudinally assessed in 30 randomly selected larvae per cohort at 48, 72, 96, and 120 hpf using a Nikon SMZ18 stereomicroscope (Tokyo, Japan). Total abnormality rates incorporating both morphological defects and mortality were systematically quantified. Subsequent analyses specifically quantified both mortality rates and malformation prevalence at the 72 hpf developmental stage. Cardiac rhythm analysis was performed using a Nikon A1+ confocal imaging system (Tokyo, Japan), with heart rate quantified. All experimental procedures were conducted through three independent technical replicates and biological replicates. Digital image analysis was performed using ImageJ v1.50 software.

### 4.4. Detection of Reactive Oxygen Species Levels

At 8 hpf, AB/WT zebrafish embryos (20 embryos per concentration group) were treated with 0.4 µg/mL Menadione combined with gradient concentrations of Astragaloside IV (AS-IV: 36 µg/mL, 72 µg/mL, 108 µg/mL) for 3 days. A parallel control group received 0.1% DMSO vehicle solution. Zebrafish larvae were incubated with 20 μL DCFH-DA dye in 24-well plates for 1 h at 28.5 °C in the dark. Ten larvae were selected for fluorescence microscopy (excitation filter wavelength 488 nm) to observe fluorescence intensity. The experiment was performed with three biological replicates.

### 4.5. Acridine Orange (AO) Staining

At 3 days after treatment with 0.4 µg/mL Menadione combined with gradient concentrations of AS-IV (36 µg/mL, 72 µg/mL, 108 µg/mL) for 24 hpf zebrafish embryos, 10 embryos per group were used for AO staining. Zebrafish were exposed to a 7 μg/mL concentration of the AO probe for a duration of 20 min. Subsequently, the specimens were thoroughly rinsed multiple times with phosphate-buffered saline (PBS). The fluorescence intensity of the zebrafish larvae was observed using fluorescence microscope (Nikon, SMZ18, Tokyo, Japan) (excitation filter wavelength 488 nm). In this experimental set up, the acquired data were meticulously measured by Image J v1.50. The experiment was performed with three biological replicates.

### 4.6. Real-Time Quantitative PCR Analysis

At 8 hpf, AB/WT zebrafish embryos (40 per concentration group) were co-treated with 0.4 μg/mL Menadione and graded concentrations of AS-IV (36, 72, 108 μg/mL) for 72 h. A vehicle control group received parallel treatment with 0.1% DMSO solution under identical experimental conditions. Total RNA was extracted from 40 embryos using the Trizol method. Synthesize cDNA using the Livning^®^ qPCR RT kit (Beijing, China), which includes a gDNA remover component. Real-time fluorescence quantitative PCR was conducted utilizing a 2X Livning SYBR Premix EsTaq plus (enriched with Tli RNaseH) (Beijing, China) on an AB Fast Real-Time PCR System, a real-time quantitative PCR instrument from ABI (Applied Biosystems, Foster City, CA, USA). The experiment was performed with three technical parallels and biological parallels.

### 4.7. Western Blot Assay of Proteins

At 8 hpf, AB/WT zebrafish embryos (50 per concentration group) were co-exposed to 0.4 μg/mL Menadione and AS-IV (36, 72, 108 μg/mL) for 72 h. A parallel control group received 0.1% DMSO vehicle solution. After removal of the soft yellow bursa from the juvenile fish at 72 hpf, RIPA lysate containing protease inhibitors was added, and the embryos were ultrasonically fragmented until they were completely cleaved as total proteins, after which they were subjected to SDS-page electrophoresis, membrane transfer, antibody incubation, and ECL development. Primary antibodies against the following targets were used: γH2AX (10856-1-AP, 1:1000), cleaved caspase-3 (19677-1-AP, 1:1000), NRF2 (80593-1-RR, 1:500), phospho-AKT (8731-1-AP, 1:3000), and KEAP1 (10503-2-AP, 1:2000). All antibodies were purchased from ProteinTech Group (Wuhan, China). The experiment was performed with three biological replicates.

### 4.8. Web-Based Pharmacology Analysis

The following resources were used: ETCM (Encyclopedia of Traditional Chinese Medicine) (http://www.tcmip.cn/ETCM/, accessed on 11 November 2024); TCMSP database (traditional Chinese medicine systems pharmacology database and analysis platform) (https://old.tcmsp-e.com/, accessed on 11 November 2024); HERB Herbal Histology Database (http://herb.ac.cn/, accessed on 11 November 2024); GeneCards database (https://www.genecards.org/, accessed on 11 November 2024); UniProt database (https://www.genecards.org/, accessed on 11 November 2024); David database (https://david.ncifcrf.gov/home.jsp, accessed on 11 November 2024); STRING database (https://cn.string-db.org/, accessed on 11 November 2024); VENNY2.1 database (https://bioinfogp.cnb.csic.es/tools/venny/, accessed on 11 November 2024); KEGG database (Kyoto Encyclopedia of Genes and Genomes) (https://www.genome.jp/kegg/, accessed on 12 November 2024); Microbiotics mapping platform (http://www.bioinformatics.com.cn/, accessed on 11 November 2024).

### 4.9. Statistical Analysis

All data were statistically analyzed using the GraphPad Prism 10 software (San Diego, CA, USA) with a two-tailed Student’s *t*-test. Statistical significance was set at *p* < 0.05. Error values were presented as the standard error of the mean (SEM). Each experiment was repeated at least thrice.

## 5. Conclusions

Menadione can induce oxidative stress injury in zebrafish. One of the most important aberrant phenotypes is zebrafish pericardial edema. Reactive oxygen species will induce DNA damage and cell apoptosis. AS-IV can inhibit apoptosis by activating the NRF2 signaling pathway and eliminating ROS thereby reducing DNA damage. Therefore, we verified that AS-IV has a therapeutic effect on developmental toxicity caused by oxidative stress in zebrafish. AS-IV has been shown to inhibit cellular apoptosis and damage phenotype in zebrafish oxidative stress models by regulating the AKT/NRF2/OH-1/caspase-3 axis. The results of this study will help us to understand the molecular mechanism of AS-IV antioxidant and will provide an experimental basis for the modernization of Chinese medicine.

## Data Availability

All relevant data are included in this article and presented as figures, tables, or Supplementary Materials. Other data are available from the corresponding author upon reasonable request.

## Supplementary Materials

The following supporting information can be downloaded at: https://www.mdpi.com/article/10.3390/molecules30112355/s1. Figure S1: AS-IV toxicology experiment. (A) Effects of AS-IV on zebrafish mortality; (B) effects of AS-IV on zebrafish deformity rates. Figure S2: Assay of γH2AX, cleaved caspase-3, NRF2, KEAP1, and *p*-AKT content by Western blot analysis showed that AS-IV inhibited oxidative stress.

## Abbreviations

The following abbreviations are used in this manuscript:

AKT/PKB	Protein kinase B
AS-IV	Astragaloside IV
AO	Acridine Orange 10-nonyl bromide
ARE	Antioxidant response element
CAT	Antioxidant response element
DMSO	Dimethyl sulfoxide
DCFH-DA	2′-7′dichlorofluorescein diacetate
DAVID	Database for Annotation, Visualization, and Integrated Discovery
GPX	Glutathione peroxidase
GO	Gene Ontology Enrichment Analysis
HO-1	Heme oxygenase-1
Keapl	Kelch-like ECH-associated protein 1
KEGG	Kyoto Encyclopedia of Genes and Genomes
MEN	Menaquinone
NRF2	Nuclear factor erythroid 2-related factor 2
OS	Oxidative stress
PPI	Protein–protein interaction
ROS	Reactive oxygen species
SOD	Superoxide dismutase
SPSS	Statistical Package for the Social Sciences
