# Supplementary material for: AS-IV Attenuates Oxidative Stress-Induced Apoptosis in Zebrafish via Modulation of the AKT/NRF2/HO-1/Caspase-3 Signaling Axis
Source: Molecules. 2025 May 28;30(11):2355. doi: 10.3390/molecules30112355 (PMC12155876; doi:10.3390/molecules30112355)
Supplement: Supplementary file 1 [file molecules-30-02355-s001.zip › molecules-3591702-supplementary.pdf]

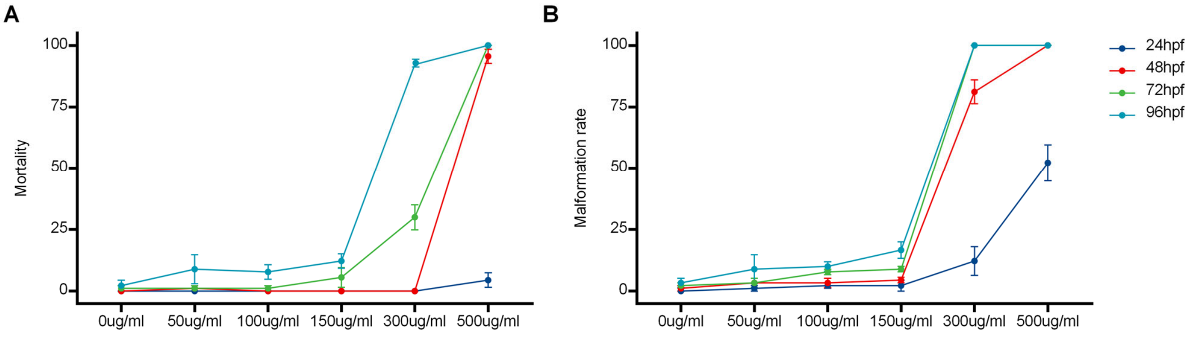

Figure S1. AS-IV toxicology experiment. (**A**) Effects of AS-IV on zebrafish mortality; (**B**) effects of AS-IV on zebrafish deformity rates.

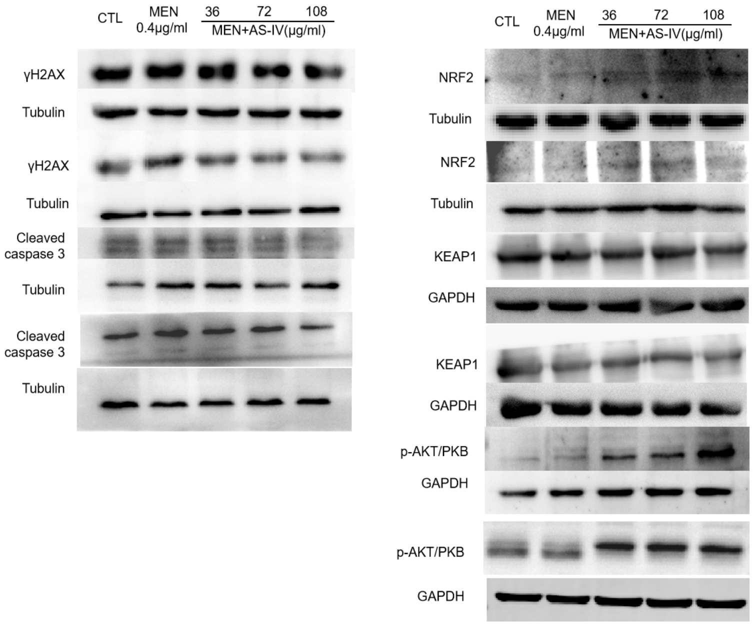

Figure S2. Assay of γH2AX, cleaved caspase-3, NRF2, KEAP1, and *p*-AKT content by Western blot analysis showed that AS-IV inhibited oxidative stress.
